# Everyday Digital Support to Promote Health and Literacy Among Older Adults: 14-Week Randomized Digital Pilot Trial by Engagement Level

**DOI:** 10.2196/77319

**Published:** 2025-12-10

**Authors:** Andressa Crystine da Silva Sobrinho, Guilherme da Silva Rodrigues, Guilherme Lima de Oliveira, Grace Angélica de Oliveira Gomes, Carlos Roberto Bueno Júnior

**Affiliations:** 1 Department of Clinical Medicine, Ribeirão Preto Medical School, University of São Paulo (FMRP-USP), 3900 Avenida Bandeirantes, Ribeirão Preto, São Paulo, 14049-900, Brazil, 55 16988155152; 2 Ribeirão Preto Medical School, University of São Paulo (FMRP-USP), Ribeirão Preto, São Paulo, Brazil; 3 Department of Clinical Medicine, Ribeirão Preto Medical School, University of São Paulo (FMRP-USP), Ribeirão Preto, São Paulo, Brazil; 4 Department of Gerontology, Federal University of São Carlos (UFSCar), São Carlos, São Paulo, Brazil

**Keywords:** health promotion, patient engagement, digital health tools, behavioral change, older people care

## Abstract

**Background:**

While digital health solutions are becoming increasingly sophisticated, simple forms of everyday digital support may offer underexplored opportunities to promote health among older adults. However, evidence remains scarce on whether such teleassistance-based approaches can effectively enhance health literacy and daily self-care, particularly among populations facing socioeconomic and educational disparities.

**Objective:**

This study examined whether a 14-week mobile teleassistance intervention could support daily health promotion and improve health literacy and quality of life among older adults, and whether different levels of user engagement were associated with differences in outcomes.

**Methods:**

This randomized digital pilot study involved 21 older adults (aged ≥60 years) from Ribeirão Preto, Brazil. All participants were assigned to the intervention arm and subsequently categorized into high-engagement (n=11) and low-engagement (n=10) subgroups according to platform-use metrics. The intervention combined weekly teleconsultations, gamified educational quizzes, and guided health-related activities delivered through a mobile app. Outcomes included health literacy (Health Literacy Questionnaire), quality of life (36-Item Short-Form Health Survey), physical activity, and sedentary behavior, assessed at baseline and postintervention. Analyses appropriate for small samples were applied, including frequentist and Bayesian models.

**Results:**

Participants in the high-engagement subgroup showed greater improvements in health literacy compared with those in the low-engagement subgroup (mean change +9.5 vs +9.1 points; time × group: *P*<.001; Bayes Factors [BF₁₀]=15). Significant interactions also favored higher engagement for selected quality-of-life domains: vitality (*P*≤.001), functional capacity (*P*=.02), and general health (*P*=.02). A group effect was observed for the mental component (*P*<.001). Physical activity (*F*_2,38_=0.95; *P*=.39; BF_incl=0.68) and sedentary behavior (F_1,19_=1.12; *P*=.32; BF_incl=0.53) did not differ significantly between subgroups. Engagement analytics confirmed higher overall platform use in the high-engagement subgroup (mean 6483.8, SD 807.0 vs mean 3345.3, SD 742.7; *t*_19_=6.238; *P*<.001; d=2.73) and more weekly health-activity minutes (mean 5124.3, SD 757.9 vs mean 3120.7, SD 704.3; *t*_19_=6.256; *P*<.001; d=2.73).

**Conclusions:**

This 14-week randomized digital pilot trial suggests that everyday digital teleassistance may enhance health literacy and specific quality-of-life domains among older adults when engagement is high. However, such support alone appears insufficient to modify physical activity or sedentary behavior in the short term. Larger and longer trials are needed to assess sustainability, scalability, and strategies to address structural inequalities in digital health adoption.

## Introduction

Population aging presents significant challenges for health care systems, particularly regarding the promotion of autonomy, quality of life, and engagement in health practices among older adults [1]. Despite advancements in preventive medicine and health education, a critical gap remains in the integration of accessible digital technologies that can effectively engage and positively impact this demographic. Previous studies have demonstrated that technology-based interventions, such as health apps, can enhance health literacy and quality of life, but these often lack robust engagement strategies or usability features tailored to older adults [2,3].

Given the complexity of behavior change in later life, a conceptual framework is essential to clarify mechanisms of action and guide hypothesis testing. We posit that mobile teleassistance influences outcomes through a multistep pathway. First, the intervention enhances access to understandable and actionable information (health literacy), while reducing barriers to navigation (remote guidance) and providing autonomy-supportive prompts (gamified tasks and teleconsultations).

These elements strengthen capability, opportunity, and motivation to engage in self-care (capability, opportunity, motivation–behavior model [4]), increase self-efficacy and autonomous motivation (self-determination theory [5]), and foster patient-provider connectedness. Proximal mediators are (1) digital engagement with the platform (use intensity and adherence), (2) health literacy gains, and (3) self-efficacy for daily management. Distal outcomes include quality-of-life domains and health behaviors (physical activity and sedentary time). We further anticipate moderation by baseline digital literacy, socioeconomic position, and cognitive status, as well as usability features.

Older adults often face intersecting barriers—lower digital literacy, socioeconomic constraints, and cognitive changes—that undermine the effectiveness of otherwise promising digital tools. Previous reviews show benefits of digital health literacy interventions in later life [6,7], yet mechanistic clarity and theoretically informed models remain underexplored, particularly regarding how engagement operates as a proximal mediator of intervention effects. Engagement is frequently treated as a crude exposure rather than as a dynamic behavioral construct informed by theory and technology-use frameworks [8,9].

Moreover, few studies examine how variations in engagement within the same intervention influence outcomes. In this context, frameworks such as reach, effectiveness, adoption, implementation, and maintenance [10] emphasize reach, adoption, and fidelity as crucial for scalability but are rarely applied to understand everyday teleassistance in older populations. Addressing these gaps, this study advances the evidence base by testing a theory-informed teleassistance intervention, explicitly modeling engagement as a mediator, and considering contextual moderators relevant to real-world implementation.

Guided by this model, we evaluated a multifaceted mobile teleassistance program for adults aged 60 years and older. The primary objective was to test whether higher engagement with the platform is associated with improvements in health literacy and quality-of-life domains. Secondary objectives were to examine effects on health behaviors (physical activity and sedentary time) and to explore the mediating roles of engagement and health literacy, as well as moderation by socioeconomic and cognitive factors. Based on this framework, we formulated the following hypotheses:

1. Primary hypothesis: participants with higher engagement in the teleassistance platform would demonstrate greater improvements in health literacy and quality-of-life domains over 14 weeks compared with those with lower engagement.
2. Secondary hypothesis: changes in physical activity and sedentary behavior could occur, but were expected to be modest in the absence of a structured exercise component.
3. Mechanistic hypothesis: engagement and health literacy would function as proximal mediators of intervention effects, whereas socioeconomic and cognitive factors would moderate these associations.

By addressing these conceptual and empirical gaps, this study contributes to advancing the evidence base on everyday digital teleassistance for older adults, offering insights that may inform the development of more scalable and context-sensitive digital health strategies.

## Methods

### Trial Design

This was a randomized, controlled, single-blind trial with allocation concealment and blinded outcome assessment, conducted in Ribeirão Preto, São Paulo, Brazil, between July and December 2023. A total of 21 community-dwelling adults aged 60 years and older were enrolled via convenience sampling through social and local media and community outreach (including the Bom Prato initiative). Inclusion criteria were ownership of a mobile device capable of supporting the teleassistance app and absence of severe visual, hearing, or cognitive impairments. Cognitive function was screened using the Montreal Cognitive Assessment (MoCA [11,12]). Randomization (1:1) to the intervention (app) or control was performed by an independent researcher.

### Control Group Condition

Participants allocated to the control group did not receive the app or any alternative intervention during the 14-week study period; they continued their usual daily routines and standard health care access. At the end of the study, control participants were invited to voluntarily participate in the teleassistance intervention, ensuring equitable access after trial completion.

### Rationale for Duration

The 14-week duration was selected in line with previous digital health literacy and teleassistance interventions in older adults [11-13], which commonly span 8‐16 weeks, and to allow adequate time for habit formation and perception of healthier daily routines, while balancing feasibility and retention in community settings.

For the present analyses, we focused on participants allocated to the app group, who were subsequently subdivided exploratorily into high- and low-engagement subgroups according to platform use. The final analytic sample comprised 11 participants in the higher-engagement subgroup and 10 in the lower-engagement subgroup, meeting a priori power-analysis requirements.

### Study Type

This investigation is part of a comprehensive project evaluating a multifaceted mobile teleassistance app for older adults [11]. The app had been previously validated for usability and effectiveness [13], supporting its use in this trial. It integrates functionalities developed by a multidisciplinary team (physicians, nutritionists, nurses, physical education professionals, and psychologists) to promote health and support healthy aging. The overarching project adopts an integrated quantitative approach using a randomized controlled design with 2 main groups (control and intervention [11]). In this paper, we specifically analyze the app group over 14 weeks, with engagement-based subdivision derived from platform interaction metrics (eg, frequency of access and participation in activities). Gamification was used to motivate participation and to classify users into higher- and lower-score categories.

**Figure 1.**
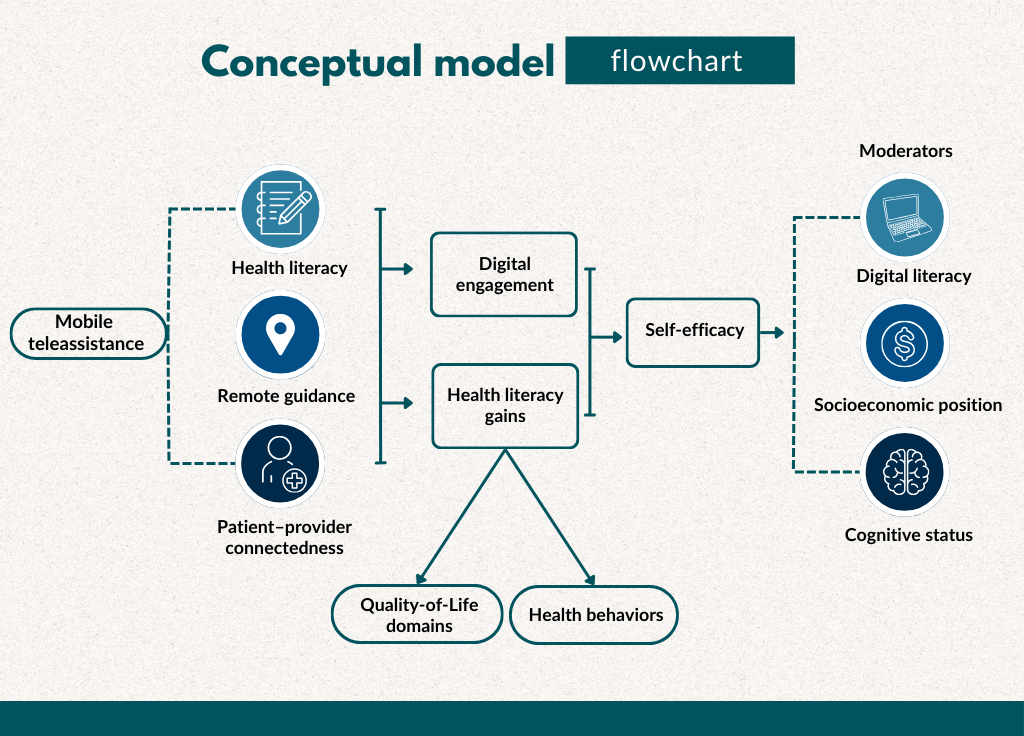
Conceptual model outlining the hypothesized mechanisms through which mobile teleassistance influences health literacy and health behaviors among older adults.

Figure 1 illustrates the theoretical model underpinning the intervention and analyses, depicting how engagement with teleconsultations, educational quizzes, and guided activities was hypothesized to influence health literacy, daily practices, and selected quality-of-life domains, moderated by factors such as digital literacy, socioeconomic position, and cognitive status. The model illustrates how engagement with teleconsultations, educational quizzes, and guided activities may affect health literacy, daily practices, and selected quality-of-life domains. It also considers moderating factors, such as digital literacy, socioeconomic position, and cognitive status. This conceptual model guided the study design, analytical approach, and interpretation of engagement as both an exposure variable and a proximal mediator.

### Participants and Setting

The study took place in Ribeirão Preto (SP), Brazil, with recruitment through social media, television, radio, University of São Paulo channels, and governmental initiatives such as Bom Prato. Strategies aimed to reduce selection bias and ensure socioeconomic and educational diversity, consistent with the exploratory nature of this pilot trial. Although not designed for population representativeness, this heterogeneity strengthens the external relevance of the findings.

### Intervention

Participants in the intervention arm could freely access educational quizzes, teleconsultations, and guided health-related activities through the mobile app. Teleconsultations were requested via in-app messaging and typically received responses from health care professionals within approximately 20 minutes. The app implemented a scoring system based on task completion to encourage adherence and enable engagement analytics [11]. To reduce technological barriers, structured technical support was provided daily during the first week, every 3 days during the following 2 weeks, and biweekly thereafter, with on-demand assistance available throughout the intervention [11]. This approach enabled assessment of both the impact of intensive app use and differences associated with engagement levels. Control participants received the app offer after the study, as detailed in the “Control Group Condition” section.

### Participants (Eligibility, Randomization, and Subgrouping)

#### Eligibility

Individuals aged 60 years and older who owned a compatible mobile device were eligible; the device requirement was pivotal because the intervention depended on app use. Exclusion criteria included conditions that would preclude study assessments (eg, severe visual, hearing, or cognitive impairments). The MoCA [12] was administered to characterize cognitive function and to contextualize any difficulties in using the app.

#### Randomization and Allocation

Random allocation (1:1) to app or control groups was conducted by a blinded, independent researcher using the random number generator in Microsoft Excel 2013, with age bands (60‐65 and 66‐70 years) considered to enhance balance.

#### Subgrouping

After the start of the intervention, an exploratory secondary allocation within the app group divided participants into high- and low-engagement subgroups using the median of total engagement scores as the cutoff [14,15]. The subdivision was automated to minimize investigator influence. Exploratory checks of baseline equivalence (eg, age, socioeconomic level, and years of education) between engagement subgroups were performed [16].

### Tests and Assessments

Cognitive status was screened with the MoCA [12] to characterize the sample and contextualize usability. Physical activity was measured with the International Physical Activity Questionnaire (IPAQ), classifying participants as low, moderate, or high activity [17]. Sedentary behavior was assessed by self-reported average daily sitting time, supplemented by IPAQ items specific to sedentary behavior [18].

Health literacy was assessed with the Test of Health Literacy for Portuguese-speaking Adults (THL-PA [19]), capturing comprehension and management of health-related information. Quality of life was measured with the 36-Item Short-Form Health Survey (SF-36), encompassing physical, emotional, social, and mental health domains [20].

The app automatically recorded the number of quizzes accessed, teleconsultations completed, and weekly time dedicated to health-related activities (eg, personal self-care, accessing health services such as pharmacies, participation in religious practices). These indicators supported classification into higher versus lower engagement for comparative analyses. Socioeconomic status was categorized using the Brazilian Socioeconomic Classification Questionnaire [21]. Demographic variables (eg, age and years of education) were collected by trained researchers to ensure standardization and reliability.

Weight, height, and physical performance tests were collected at baseline and postintervention to contextualize potential physical changes, as described previously [11,13]. Primary endpoints were changes in health literacy (THL-PA) and quality of life (SF-36). Secondary endpoints were physical activity, sedentary behavior, and engagement metrics (eg, quizzes accessed and teleconsultations).

### Sample Size

A priori estimation using G*Power (version 3.1; Franz Faul, Edgar Erdfelder, Axel Buchner, and Albert-Georg Lang; University of Kiel and University of Mannheim) for repeated-measures ANOVA assumed a medium effect size (f=0.25), α=.05, power=0.80, 2 time points (pre and post), 2 groups (high vs low engagement), and a within-subject correlation of 0.50, based on conservative literature [22]. The required total sample was 20 (10 per group). The final analytic sample comprised 21 (high engagement=11 and low engagement=10), exceeding the minimum and ensuring adequate model stability.

### Statistical Analysis

Data were organized in Microsoft Excel and analyzed using SPSS (version 20; IBM Corp) and Jeffreys’s Amazing Statistics Program (JASP; version 0.19; JASP Team; University of Amsterdam). Descriptive statistics included means, SDs, and percentages, with normality tested by the Kolmogorov-Smirnov test (Lilliefors correction).

Given the small sample (N=21), both frequentist and Bayesian methods were applied. Bayesian analyses (JASP) provided posterior probabilities and Bayes Factors (BF₁₀) to quantify evidence for the alternative hypothesis over the null hypothesis (BF₁₀>3=moderate, >10=strong, and >30=very strong).

Between-group differences in continuous variables were tested using independent-samples *t* tests, and categorical variables were tested using 2-sided Fisher exact tests. Longitudinal outcomes were examined with a generalized linear model and generalized linear mixed model (GLMM), specifying group (high vs low engagement) as a fixed factor and time (pre vs post) as repeated.

Model fit was assessed using Akaike information criterion, Wald *χ*², and 95% CIs for β coefficients, with effect sizes (Cohen *d*, η²). Bayesian repeated-measures ANOVA provided BF for inclusion (BF_incl). Sensitivity analyses excluded very-low-engagement participants (<20%) and outliers (|z|>3.29), yielding consistent results.

Missing data (<5%) were imputed by chained equations (multivariate imputation by chained equations and missing at random assumption) and pooled via Rubin’s rules. Bonferroni corrections adjusted the Type I error, and both raw and adjusted *P* values are reported. Visualizations (raincloud and Flexplots) were generated in JASP. Statistical significance was set at *α*=.05 (2-sided).

### Ethical Considerations

The study was conducted by the lead researcher (ACdSS, Health Sciences) at the Ribeirão Preto Medical School of the University of São Paulo (FMRP/USP). All procedures complied with Resolution 466/2012 of the Brazilian National Health Council and adhered to international bioethical principles. The protocol was approved by the Research Ethics Committee of the Ribeirão Preto School of Physical Education and Sport, University of São Paulo (process no 58433922.0.0000.5659; October 26, 2022). All participants received detailed information about the study objectives, procedures, potential risks, and benefits and provided written informed consent before participation. Confidentiality was ensured through anonymized data handling, secure digital storage, and restricted access. Data were used exclusively for academic and scientific purposes. The trial was registered in the Brazilian Clinical Trials Registry (ReBEC: RBR-6wgkzs8; “Development of an Application to Improve Health in Older People”). Participants did not receive any financial compensation or material incentives for participation.

## Results

### Sample Characteristics

The final sample comprised 21 participants divided into 2 groups: the higher-score group (higher, n=11) and the lower-score group (lower, n=10). The mean age in the higher group was 64.73 (SD 3.23) years, while the lower group had a mean age of 63.10 (SD 2.99) years. Normality was confirmed using the Kolmogorov-Smirnov test (*P*>.05). Independent *t* tests showed no significant differences between groups (*t*_19_=1.20; *P*=.25), corroborated by Bayesian results (BF₁₀=0.59), indicating anecdotal evidence in favor of the null hypothesis.

### Socioeconomic and Educational Characteristics

According to the Brazilian socioeconomic classification criteria, the higher group had a mean score of 26.55 (SD 10.42), while the lower group scored 32.60 (SD 8.33). The Fisher exact test indicated no statistically significant differences in category distribution (*P*=.45). Bayesian contingency analysis provided moderate evidence supporting the null hypothesis (BF₁₀=0.41). Regarding education, participants in the higher group reported an average of 11.0 (SD 2.8) years of formal schooling, compared with 11.7 (SD 3.7) years in the lower group. Independent-samples *t* test results showed no significant between-group differences (*t*_19_=−0.49; *P*=.63), and Bayesian analysis indicated anecdotal evidence supporting the null hypothesis (BF₁₀=0.45). These results suggest that the groups were comparable in terms of age, education, and socioeconomic background at baseline.

### Physical Activity and Sedentary Behavior

Physical activity levels, assessed using the IPAQ, were classified as low, moderate, or high. Preintervention, 45.5% of the higher group had low activity, 45.5% moderate, and 9.1% high. In the lower group, 50% were low and 50% moderate. Postintervention, 27.3% of the higher group had low activity, 45.5% moderate, and 27.3% high. The lower group had 20% low, 30% moderate, and 50% high.

To analyze differences over time, a GLMM was conducted with physical activity level as the dependent categorical variable, group as a fixed factor, and time as a repeated measure. No significant interaction was found (*F*_2,38_=0.95; *P*=.39), and Bayesian GLMM results yielded a BF_incl=0.68, indicating only anecdotal evidence for a group-by-time effect. Sedentary behavior, measured as sitting time, showed that before the intervention, 90.9% of the higher group and 100% of the lower group were highly sedentary. After the intervention, the distribution remained unchanged in the higher group, while 20% of the lower group shifted to moderate sedentary levels. GLMM analysis revealed no significant group-by-time interaction for sedentary behavior (*F*_1,19_=1.12; *P*=.32), with a Bayesian BF_incl=0.53, again indicating anecdotal support for the null hypothesis.

### Health Literacy and Quality of Life (SF-36)

Table 1 displays the outcomes for health literacy and SF-36 quality-of-life domains. Both groups improved significantly in health literacy, with the higher group exhibiting a greater gain (interaction T×G: *P*<.001; BF₁₀=15.2, indicating strong evidence in favor of a group-by-time effect). For the physical domain, a significant postintervention reduction was noted in the lower group (T×G: *P*=.002; BF₁₀=10.4), suggesting strong evidence for change. The mental domain remained stable over time but differed significantly between groups (group effect: *P*<.001; BF₁₀=12.7). Values are presented as mean and SD. Post hoc analyses include main effects of time, group, and their interaction (T × G), Wald statistics, *P* values, standardized beta coefficients, 95% CIs, and Akaike information criterion (AIC) values. Variables assessed include health literacy and multiple SF-36 domains (physical, mental, general health, functional capacity, vitality, physical aspects, emotional aspects, social aspects, mental health, and pain). Significant effects are indicated at *P*<.05. “Time” reflects pre-post changes across both groups, “group” reflects between-group differences, and “T × G” represents the interaction between time and group.

**Table 1.** Pre- and post-intervention comparisons between engagement groups.

Variable	Higher-score group, mean (SD)	Higher-score group, mean (SD)	Lower-score group, mean (SD)	Lower-score group, mean (SD)	Post hoc (*F*,*P* value)			Wald test	*P* value	ß coefficients (95% CI)	AIC^a^
	Preintervention	Postintervention	Preintervention	Postintervention	Time	Group	T×G				
Health literacy	80.6 (15.6)	90.1 (13.7)	72.5 (17.5)	81.6 (12.9)	4.1, .04	3.4, .07	7.7, <.001	2.1	.03	75.9 (66.3‐406.7)	342.7
Physical domain	51.9 (10.4)	50.2 (9.9)	61.9 (7.8)	57.6 (8.3)	1.0, .30	9.4, .<001	1.3, <.001	3.1	.002	83.1 (44.4‐155.3)	301.9
Mental domain	41.5 (9.4)	41.5 (9.4)	54.4 (10.3)	54.4 (10.3)	0.0, ≥.99	18.2,<.001	9.2, ≥.99	3.1	.002	94.6 (50.7‐176.5)	306.5
General health status	14.2 (3.6)	13.4 (3.0)	19.9 (2.8)	16.2 (2.0)	2.9, .30	8.5, .20	8.0, .02	2.1	.03	4.04 (1.6‐10.1)	215.4
Functional capacity	23.3 (5.1)	22.2 (5.1)	26.7 (2.5)	25.4 (3.0)	1.3, .26	5.8, .02	5.2, .02	2.1	.03	9.2 (3.7‐22.9)	237.5
Vitality	13.6 (5.0)	13.6 (5.0)	16.6 (2.9)	16.6 (2.9)	0.0, ≥.99	5.4, .02	7.7, .<.001	3.1	.002	16.8 (9.0‐31.4)	239.3
Physical aspect	6.6 (1.3)	6.6 (1.3)	6.5 (2.0)	6.5 (2.0)	0.0, .70	0.0, .04	0.0, .07	2.1	.02	3.9 (1.5‐9.5)	170.0
Emotional aspect	4.0 (1.3)	4.0 (1.3)	5.0 (1.3)	5.0 (1.3)	0.0, ≥.99	0.0, ≥.99	0.0, ≥.99	2.1	.30	1.72 (0.7‐4.2)	153.5
Social aspect	7.0 (1.9)	7.0 (1.9)	8.3 (1.5)	8.3 (1.5)	0.0, ≥.99	5.0, .03	0.0, ≥.99	3.1	.02	3.0 (1.6‐5.6)	172.2
Mental health	18.9 (4.3)	18.9 (4.3)	22.1 (3.3)	22.1 (3.3)	0.0, ≥.99	11.2, <.001	30.8,<.001	3.1	.002	14.9 (8.0‐27.9)	234.6
Pain	7.0 (2.4)	7.0 (2.4)	8.4 (2.3)	8.4 (2.3)	0.0, ≥.99	3.6, .06	3.6, .06	2.1	.30	5.5 (2.2‐13.6)	200.5

a AIC: Akaike information criterion.

Other domains—general health, functional capacity, and vitality—also showed significant improvements, with BF ranging from 4.3 to 9.8, indicating moderate to strong evidence. In contrast, physical, emotional, and social aspects remained stable (BF₁₀<1). Mental health showed a greater postintervention increase in the lower group (T×G: *P*<.001; BF₁₀=17.6), indicating very strong evidence for group-specific effects. These findings reinforce the positive impact of the intervention, especially in promoting improvements in health literacy and selected quality-of-life domains, with Bayesian analysis enhancing confidence in the observed effects despite the small sample size.

**Figure 2.**
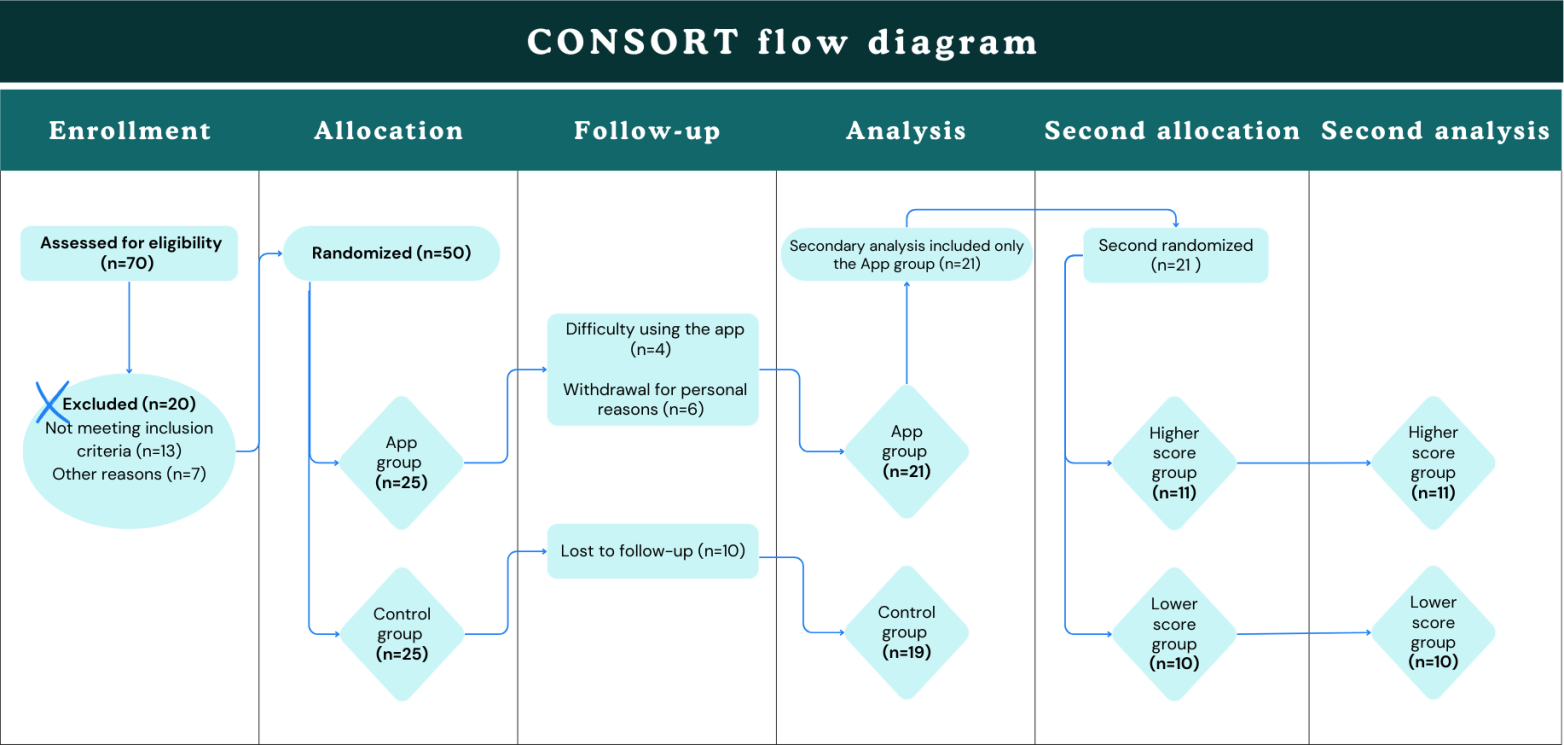
Flow of participants through the study. This randomized digital pilot trial involved older adults (≥60 years) from Ribeirão Preto, Brazil. Participants were randomized to an intervention (mobile teleassistance app) and a control group. The intervention lasted 14 weeks and combined teleconsultations, gamified educational quizzes, and guided health activities. Secondary analyses focused on the 21 participants in the intervention arm, who were further categorized into high- and low-engagement subgroups based on platform usage. Secondary allocation into high- and low-engagement subgroups was performed only for participants in the app group (N=21). The control group was not included in this exploratory analysis.

Table 2 and the raincloud plots (Figure 2) provide key metrics analyzed to compare the higher- and lower-score groups. These metrics include total engagement scores, number of quizzes accessed, number of teleconsultations performed, and average weekly time dedicated to health-related activities. Values are presented as mean and SD. Between-group differences were assessed using independent *t* tests. Metrics include total engagement score (arbitrary units), total quizzes accessed (n), number of teleconsultations (n), and total weekly minutes of health-related activities. Effect sizes are reported as Cohen *d* with 95% CIs. Significance level set at *P*<.05.

**Table 2.** Comparison of key engagement and health metrics between high- and low-scoring groups during the 14-week teleassistance intervention (Ribeirão Preto, Brazil, 2023).

Variable	Higher-score group, mean (SD)	Lower-score group, mean (SD)	*t* test (*df*)	*P* value	Effect size, *d* (95% CI)
Total score (au^a^)	6483.773 (807.034)	3345.272 (742.684)	6.238 (19)	<.001	2.726 (1.494 to 3.922)
Total quizzes accessed (n)	33.45 (3.085)	35.20 (3.372)	−0.383 (19)	.71	−0.167 (−1.023 to 0.693)
Teleconsultations (n)	14.33 (4.144)	10.00 (5.007)	0.831 (19)	.42	0.363 (−0.506 to 1.223)
Weekly health activity (minutes)	5124.273 (757.892)	3120.700 (704.249)	6.256 (19)	<.001	2.733

a au: arbitrary unit.

The total engagement score showed a substantial difference between groups: the higher-score group had a mean of 6483.77 (SD 807.03), while the lower-score group had a mean of 3345.27 (SD 742.68). The difference was statistically significant (*t*_19_=6.238; *P*<.001) with a very large effect size (Cohen *d*=2.726, 95% CI 1.494‐3.922). Bayesian *t* test results strongly supported this difference (BF₁₀=97.4), providing very strong evidence for the alternative hypothesis. Similarly, the average weekly time dedicated to health activities was significantly greater in the higher-score group (mean 5124.27 and SD 757.89 minutes) than in the lower-score group (mean 3120.70 and SD 704.25 minutes). This difference was also highly significant (*t*_19_=6.256; *P*<.001), with a very large effect size (Cohen *d*=2.733, 95% CI 1.500‐3.931), and was strongly supported by Bayesian analysis (BF₁₀=102.8).

In contrast, no significant differences were observed for the total number of quizzes accessed (*t*_19_=−0.383; *P*=.71; Cohen *d*=-0.167; BF₁₀=0.34) or the number of teleconsultations performed (*t*_19_=0.831; *P*=.42; Cohen *d*=0.363; BF₁₀=0.48). These Bayesian results indicate moderate evidence in favor of the null hypothesis, suggesting that these variables were not key contributors to differentiating user engagement levels.

**Figure 3.**
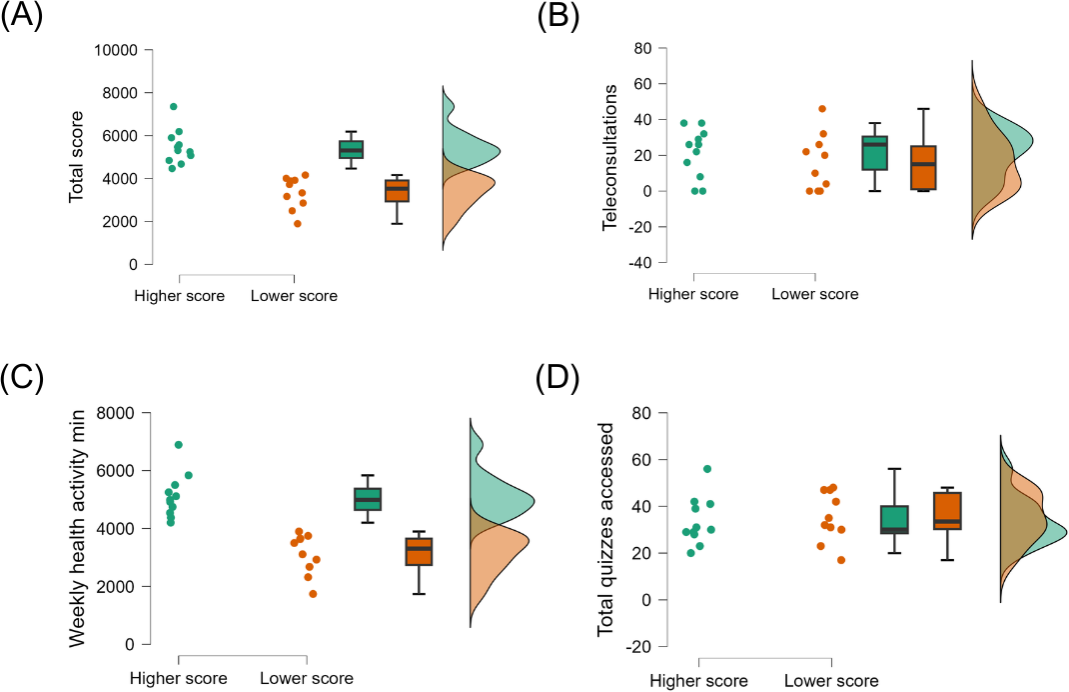
Distribution of (A) total engagement score, (B) number of teleconsultations, (C) weekly health activity minutes, and (D) total number of quizzes accessed among older adults (N=21) in the high- and low-engagement subgroups during a 14-week randomized digital pilot trial in Ribeirão Preto, Brazil. Values are represented as mean and SD. No statistically significant differences were observed at baseline between the higher- and lower-engagement groups.

The raincloud plots (Figure 3A-D) provide a detailed visualization of the distribution, variability, and central tendencies of the key variables analyzed. These plots corroborate the inferential statistics by clearly illustrating the marked differences in total engagement scores and average weekly time dedicated to health activities between the higher- and lower-score groups. By integrating raw data points, probability density estimates, and mean values, these visualizations reinforce the interpretation of the findings and offer a complementary perspective to the quantitative results.

**Figure 4.**
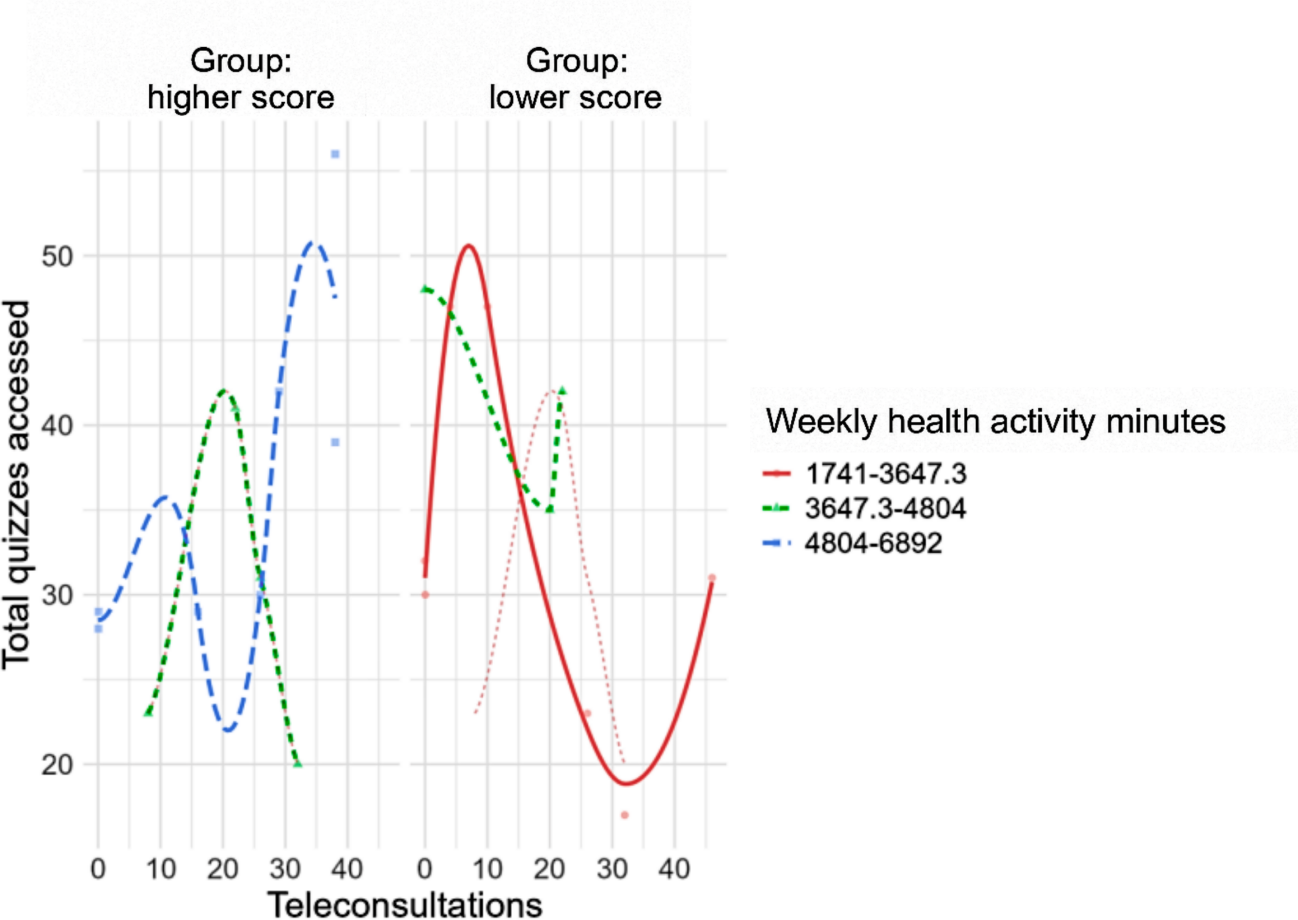
Relationship between the number of teleconsultations and total quizzes accessed, stratified by weekly health activity minutes, among older adults in the high- and low-engagement subgroups (N=21) during a 14-week randomized digital pilot trial in Ribeirão Preto, Brazil. Lines represent linear fits and shaded areas indicate 95% CIs. Patterns illustrate differential engagement behaviors across groups.

The Flexplot visualizations (Figure 4), generated using JASP software, were used to explore interaction patterns between engagement-related variables across groups. These plots merge individual-level data with modeled trends, facilitating intuitive identification of between-group differences and variability within groups.

For the total engagement score, the Flexplot revealed a clear and consistent separation between the two groups, with the higher-score group exhibiting both higher mean values and lower within-group dispersion, indicating greater uniformity and coherence in engagement. Similarly, the plot for weekly time dedicated to health-related activities demonstrated a broader spread of values overall but with a notable rightward shift in the higher-score group, reflecting more intensive engagement over time (Figure 4).

**Figure 5.**
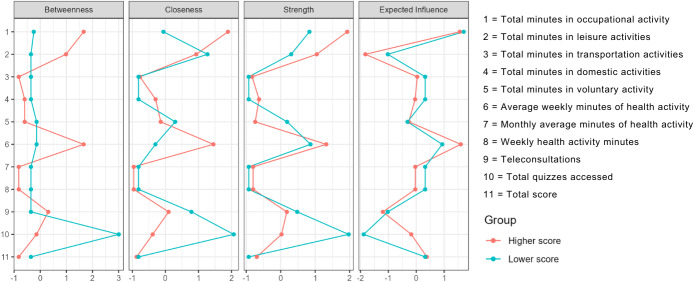
Network centrality metrics (betweenness, closeness, strength, and expected influence) for 11 behavioral and digital engagement indicators in older adults (N=21) participating in a 14-week randomized digital pilot trial in Ribeirão Preto, Brazil. Variables: 1=occupational activity, 2=leisure activity, 3=transportation activity; 4=domestic activity, 5=voluntary activity, 6=average weekly health activity, and 7=monthly health activity, 8=weekly health activity, 9=teleconsultations, 10=quizzes, and 11=total score. Node size is proportional to strength, and edges represent partial correlations between variables. Higher centrality values indicate greater influence within the engagement-behavior network.

The centrality graph (Figure 5) complements the previous findings by illustrating the relative influence of each variable within the network of relationships observed in the dataset. It identifies total engagement score and weekly time dedicated to health-related activities as the most central metrics, indicating their dominant role in distinguishing between the higher- and lower-score groups. These variables show stronger interconnections with other dimensions analyzed, reinforcing their relevance to the intervention’s overall impact.

In addition, the graph highlights secondary variables, such as the number of teleconsultations performed, which exhibit lower centrality but still contribute meaningful contextual information. Although these variables have less direct influence on group differentiation, their inclusion enhances the multivariate understanding of engagement patterns and behavioral responses to the intervention (Figure 4).

These graphical representations, alongside the statistical analyses, provide a comprehensive and multidimensional view of the relationships among the studied variables and between the comparison groups. They not only corroborate the quantitative findings but also offer valuable visual insights into the internal dynamics of the dataset, thereby enhancing the robustness, interpretability, and depth of the study’s conclusions.

## Discussion

### Principal Findings

The results of this study highlight associations between the proposed intervention and improvements in health literacy and selected domains of quality of life, such as general health status, functional capacity, vitality, and mental health. These improvements appeared particularly notable in the high-scoring group, suggesting a potential positive influence of the intervention on physical and psychological well-being. While no significant differences in physical activity levels or sedentary behavior were observed between groups over time, the analysis indicated greater engagement with health practices in the higher-performing group, as reflected by significantly higher total scores and weekly time dedicated to health-related activities.

These associations suggest that the intervention may have contributed to improvements in specific aspects of quality of life, potentially fostering behavioral and cognitive changes that were reflected in participants’ daily lives. This finding indicates that frequent and consistent use of digital tools, combined with supportive strategies, could serve as a catalyst for positive changes in older populations. Throughout this discussion, we will explore how these findings align with broader patterns of health promotion, their potential practical applications, and the challenges of translating these data into effective and scalable strategies.

### Health Literacy and Quality of Life

The observed increase in health literacy aligns with evidence in the literature, which identifies health literacy as a key factor in promoting autonomy and self-care. Programs facilitating access to health information and its understanding are associated with reduced complications from chronic diseases and the adoption of healthy behaviors [23,24]. These conclusions are supported by research such as that by Zhang et al [24] and Ngiam et al [25]. Moreover, simple everyday questions play a crucial role as a gateway to self-care [5]. In this study, participants with higher levels of engagement tended to report greater capacity to engage in health practices. This finding may underscore the potential role of technology in strengthening ties between users and health care professionals while simultaneously addressing basic daily demands [11]. Technological support can also facilitate the search for and understanding of more complex issues, potentially promoting a broader and more efficient approach to health care [11].

### Engagement Patterns and Digital Behavior

The differences between the high- and low-scoring groups reflect not only the impact of the intervention but also the influence of contextual factors. A study on a physical activity app demonstrated that the inclusion of gamification features, such as leaderboards and status indicators, increased app usage and participants’ physical activity, highlighting the potential of gamification to promote adherence and engagement [15]. Health interventions based on gamification have also shown promising results [26,27]. For instance, in patients with coronary heart disease, gamification through smartphone apps enhanced participation in physical activities and improved autonomous motivation [26]. In addition, the personalization of goals in gamified health apps has been shown to increase engagement, particularly when goals are tailored to the users’ capacities and objectives [28]. The division of participants into high- and low-engagement groups should, however, be interpreted with caution. The median split approach was adopted as a pragmatic and exploratory strategy in this small pilot sample, consistent with methods used in previous digital engagement studies. While informative, these subgroup analyses are not theory-driven and should be considered preliminary, requiring replication in larger and more diverse cohorts.

### Barriers to Digital Inclusion

Socioeconomic and educational barriers [25] remain significant challenges to technological inclusion [25,29]. Addressing these factors is essential to ensure that the benefits of digital health interventions are equitably distributed and not restricted to socially or economically privileged populations. In older adults, these challenges are compounded by age-related factors such as lower digital literacy, cognitive decline, and apprehension toward adopting new technologies. Changing behavior through digital means tends to be more complex and less immediate for older individuals than in structured, face-to-face settings, where social interaction, real-time support, and group dynamics often enhance adherence and motivation.

### Physical Activity and Sedentary Behavior

Despite the observed improvements in health literacy and selected quality-of-life domains, results related to physical activity and sedentary behavior showed limited progression. This stagnation echoes findings from other studies, which indicate that older adults face a constellation of physical and environmental barriers that hinder behavioral change, including preexisting health conditions, fear of falling or injury, low social support, lack of motivation, restricted mobility, and economic constraints [29-32]. In addition, methodological limitations such as reliance on self-reported data may have affected measurement accuracy [32]. Future interventions could benefit from incorporating objective monitoring tools—such as pedometers, accelerometers, and in-app sensors—to capture behavioral outcomes more precisely and support individualized feedback loops.

It is important to emphasize that the absence of significant improvements in physical activity and sedentary behavior is a critical finding of this study. This result is consistent with previous systematic reviews and meta-analyses showing that digital interventions alone rarely produce meaningful changes in these behaviors among older adults, particularly when no structured exercise component is embedded in the program [33,34]. Behavior change in physical activity is complex and multifactorial, often requiring multimodal strategies that combine digital tools with personalized goal setting, supervised sessions, or community-based support. Our findings, therefore, highlight the need for hybrid or augmented models that integrate technology with direct physical activity components to effectively influence movement behaviors in this population. Furthermore, this reinforces the importance of considering null results not as failures, but as evidence pointing toward the boundaries of what digital health literacy interventions can achieve in isolation.

### Practical Implications of Digital Interventions

Another significant aspect is the practical potential of digital interventions. In this context, mobile health apps that use behavior change techniques, such as feedback and monitoring, stand out as promising tools for promoting adherence to interventions and behavioral change [35]. Recent studies underscore the importance of integrating interactive and personalized features to meet the specific needs of target populations, particularly older adults, who often face technological and engagement barriers [36]. In addition, hybrid models that combine personalized care plans with in-person workshops have demonstrated potential to strengthen older adults’ confidence in new care approaches. These models are particularly relevant in the postpandemic context, where social interaction has gained greater value [37]. The literature also indicates that hybrid interventions promote higher adherence by reconciling the convenience of digital technologies with the benefits of in-person support [37]. Personalization of content and accessibility of digital health technologies remain crucial factors for adoption by older adults [37]. Approaches integrating standard care practices with interactive and personalized elements have the potential to overcome engagement barriers and expand the impact of interventions [35,36]. This scenario highlights the continuous need for innovation and adjustment of digital interventions to maximize their reach and effectiveness, aligned with growing evidence in the literature [38].

### Limitations and Future Directions

The limitations of this study—particularly the small sample size and short intervention duration—may have affected the robustness of some outcomes. While the statistical approach adopted was sufficient for several variables, future studies with larger samples and extended follow-up periods are essential to strengthen the generalizability and precision of results across all outcomes. A larger sample would enable more robust and generalizable statistical analyses, while longitudinal studies could capture the long-term effects of digital interventions on the health and quality of life of older adults. Although participants were primarily recruited from low-income community programs, this population is representative of underserved older adults who often face barriers to digital health adoption, thus strengthening the external relevance of the findings.

Such investigations would also be essential for understanding how the sustained use of these technologies may influence adherence and clinical outcomes over time [38]. Furthermore, there is a critical need to integrate multidimensional approaches that combine behavioral, cognitive, and environmental aspects. Including factors such as social support, health education, and strategies tailored to older adults’ life contexts could amplify the potential impact of interventions [38]. For instance, addressing barriers such as resistance to technology use, economic accessibility, and cultural differences may enhance adherence and engagement. The literature also suggests that alignment between users’ objectives and the functionalities offered by digital tools is a key factor for the success of these initiatives [38].

Taken together, these findings suggest that digital interventions may represent a promising opportunity to support improvements in quality of life and well-being among older adults. However, the development of more inclusive and personalized solutions that address the contextual barriers faced by this population will be essential to maximize their impact on healthy aging. Therefore, continuous innovation, combined with a deep understanding of users’ needs, should guide the evolution of digital health technologies designed for older adults.

### Conclusion

This study highlights the potential of teleassistance to enhance health literacy, autonomy, and well-being among older adults. By integrating technology with personalized support and gamification, the intervention offered a feasible and scalable model for digital health promotion. However, no significant effects were found for physical activity or sedentary behavior, suggesting that literacy-oriented digital programs alone may be insufficient to drive behavioral change. Future research should refine such interventions using hybrid and multimodal strategies to better influence movement behaviors while ensuring inclusivity and adaptability in addressing the needs of aging populations.

## Supplementary material

10.2196/77319 Checklist 1 CONSORT-eHEALTH checklist (V 1.6.1).

## Abbreviations

BF: Bayes Factors
BF_incl: Bayes Factors for inclusion
GLMM: generalized linear mixed model
IPAQ: International Physical Activity Questionnaire
JASP: Jeffreys’s Amazing Statistics Program
MoCA: Montreal Cognitive Assessment
SF-36: 36-Item Short-Form Health Survey
THL-PA: Test of Health Literacy for Portuguese-speaking Adults
